# A Rare Case of Idiopathic Failure of a Bioprosthetic Mitral Valve

**DOI:** 10.7759/cureus.42643

**Published:** 2023-07-29

**Authors:** Samuel J Wlasowicz, Ronald Lott, Xavier C Zonna, Venkata Satish Pendela, Bipul Baibhav

**Affiliations:** 1 Internal Medicine, Lake Erie College of Osteopathic Medicine, Rochester, USA; 2 College of Medicine, Lake Erie College of Osteopathic Medicine, Erie, USA; 3 Internal Medicine, Lake Erie College of Osteopathic Medicine, Erie, USA; 4 Cardiology, Rochester Regional Health, Rochester, USA; 5 Cardiology, Sands-Constellation Heart Institute, Rochester Regional Health, Rochester, USA

**Keywords:** mitral regurgitation (mr), glass echocardiogram, bioprosthetic valve failure, bioprosthetic mitral valve, flail mitral leaflet, mitral valve replacement

## Abstract

A 65-year-old female with a significant history of two previous mitral valve replacement surgeries for mitral regurgitation was found to have severe mitral regurgitation again. She was determined to have a flail anterior mitral valve leaflet and underwent redo open sternotomy mitral valve replacement. This report serves to provide an example of an uncommon case of valve failure in an effort to alert clinicians to this potential complication.

## Introduction

Cardiac valves function as barriers to direct the flow of blood during systole and diastole. If damage and degeneration occur, this can lead to stenosis or regurgitation of blood flow. For the mitral valve, stenosis or regurgitation may limit cardiac output, leading to fatigue and shortness of breath on exertion. Chronic and prolonged mitral valve disease may also enlarge the left atrium, leading to distention and conduction abnormalities such as atrial fibrillation. In terms of management, valve repair is often preferred due to the preservation of native tissue. However, when significant structural valve damage is present due to chronic disease or calcifications, valve replacement is the definitive management [1].

Modern-day mitral valve replacement is associated with positive patient outcomes due to the quickly evolving nature of replacement technology and procedures. A variety of valves exist, including seven mechanical, six stented biological porcine, and one bovine pericardial prosthesis. Furthermore, the rapid development of transcatheter devices and endoscopic and robotic replacements has made the process of undergoing a mitral valve replacement more individualized [2]. Although newer, less invasive methods for valve replacement are rising in popularity. Recent studies have found that transcatheter mitral valve replacement is a safe and effective alternative with low postoperative valve dysfunction [3].

Mitral valve replacement mortality and morbidity are widely reported statistics that are highly patient-dependent due to factors such as age, comorbidities, follow-up, and adherence to medication. Operative mortality is associated with a 4-7% risk of death and is lower in minimally invasive approaches, and 10-year survival in both mechanical and biological valves with patient characteristics taken into account is between 50% and 60% [2]. Common postoperative complications include thromboembolism, prosthetic valve endocarditis, or paravalvular leaks. In addition, although biologic tissue valves spare the patient from necessary lifelong anticoagulation with mechanical valves, they are more susceptible to structural degeneration [2].

As most valve replacements have considerable longevity and survival, early failure of a replaced valve is rare. Our case demonstrates an early, idiopathic mitral valve failure of a St. Jude Tissue valve in a patient with a history of two prior mitral valve replacement surgeries. This case provides evidence of a rare occurrence of premature valve failure, the clinical course of the patient, and the treatment strategies employed. It is our hope that this case alerts clinicians in all specialties to the possibility of premature valve failure and the various causes of this condition.

## Case presentation

A 65-year-old female presented with a chief complaint of vomiting and diarrhea. The patient reported having multiple episodes of non-bloody vomitus and watery, non-bloody diarrhea for the past three days. She also complained of a dry, nonproductive cough with chest congestion for the past four days. She denied having any fever, shortness of breath, or dysuria. She reported having had a hysteroscopy with dilation and curettage nine days prior to the presentation. At presentation, she was hypoxic at 86% on arrival and required 2 L of oxygen via a nasal cannula. Her blood pressure was 170/75, her pulse was 129, her respirations were 24, and her temperature was 99.7 °F. The physical exam was largely unremarkable except for generalized abdominal tenderness.

Past medical history is significant for severe mitral valve prolapse with severe mitral regurgitation requiring two mitral valve surgeries in the past, hypertension, hyperlipidemia, supraventricular tachycardia, and pulmonary hypertension. In 2016, she underwent operative mitral valve repair with a 31-mm Duran Ancore annuloplasty band because of a marked prolapse of the P2 segment of the posterior mitral valve leaflet, which resulted in severe mitral valve regurgitation. In 2019, she was found to have worsening mitral regurgitation with flailing of the P2 scallop of the mitral valve. She subsequently underwent reoperative mitral valve replacement with a 27-mm St. Jude Medical epic tissue valve with left atrial appendage closure at that time.

**Figure 1:**
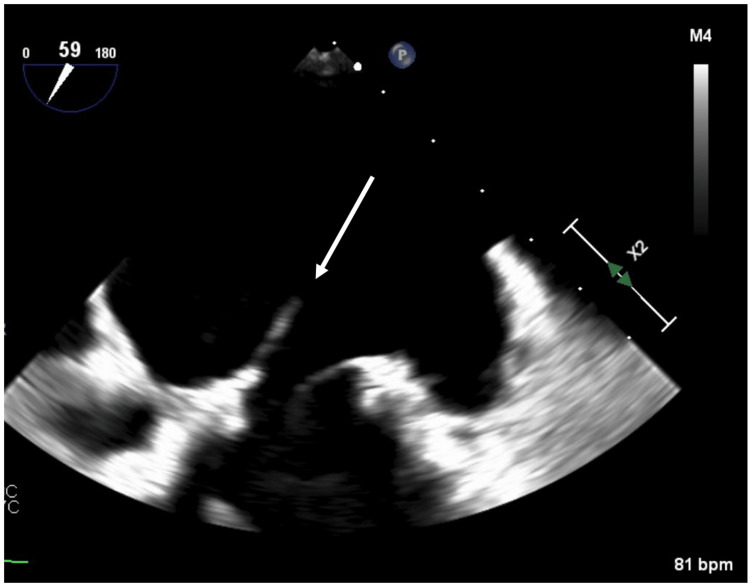
Two-chamber view with focus on mitral valve during systole. Arrow demonstrates a linear independently mobile structure attached to the mitral annulus likely representing a flail anterior leaflet which resulted in severe mitral regurgitation.

**Figure 2:**
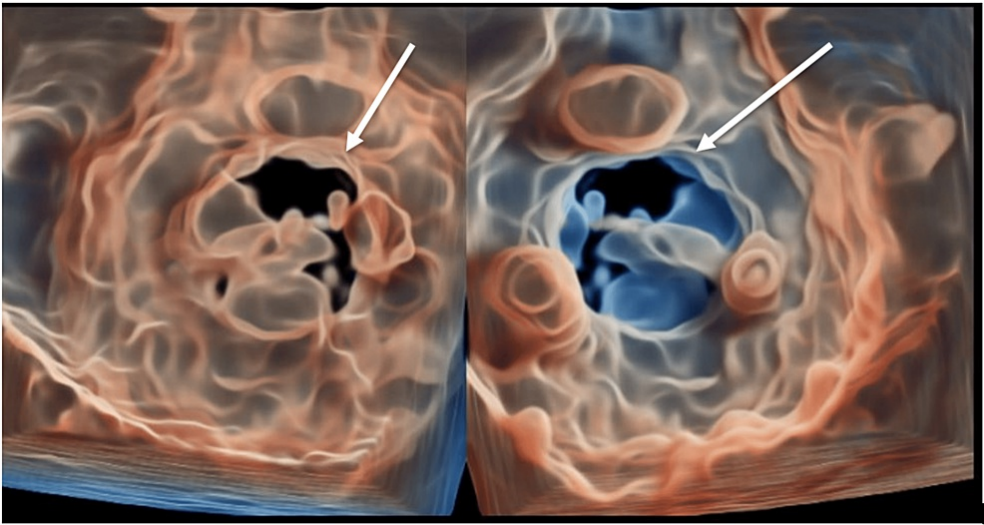
Three-dimensional “glass” reconstruction of mitral valve during systole. Arrows highlight the failure of the mitral valve to fully close due to flail anterior leaflet.

**Figure 3:**
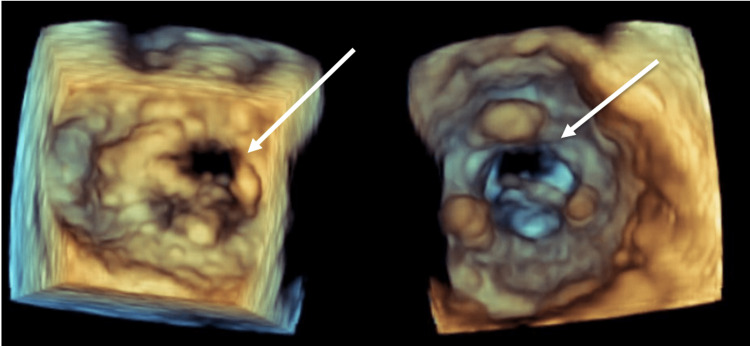
Three-dimensional reconstruction of mitral valve during systole. Arrows highlight the failure of the mitral valve to fully close due to flail anterior leaflet.

Initial labs at presentation were significant for a white blood cell count (WBC) of 15.8, a neutrophil count of 13.6, sodium of 133, and glucose of 366. Blood cultures were drawn and had no growth on preliminary results. A computed tomography (CT) scan of the abdomen and pelvis was done, which was significant for an adrenal gland lesion, a gallbladder polyp measuring 5 to 6 mm, and bilateral pleural effusions, which prompted a CT chest to be done. The CT chest was significant for bilateral alveolar disease with left upper lobe consolidation and bilateral pleural effusions. The patient was admitted to the medicine service and started on ceftriaxone and azithromycin for possible multilobular pneumonia and IV Lasix for congestive heart failure (CHF) exacerbation. Cardiology was consulted, and a transthoracic echocardiogram was done on hospital day 2, which showed a normal left ventricular ejection fraction (LVEF) of 60% and severe mitral regurgitation with systolic pulmonary vein flow reversal. No vegetation was noted on the mitral valve. A transesophageal echocardiogram (TEE) was performed, which demonstrated evidence of a linear, independently mobile structure attached to the mitral annulus (Figure 1), likely representing a flail anterior leaflet and severe mitral regurgitation. Three-dimensional reconstruction of the mitral valve using TEE imaging showed the absence of closure of one of the leaflets during systole (Figures 2-3).

Given her gastrointestinal symptoms and leukocytosis on arrival, infectious disease was consulted over concern for infectious endocarditis. Infectious disease specialists recommended continuing doxycycline and ceftriaxone and held blood cultures from her initial presentation to rule out growth from atypical organisms. Additionally, they recommended a tagged WBC scan to rule out infectious endocarditis. On hospital stay day 9, the tagged WBC scan was performed and was negative for any abnormal collection of WBCs. Blood cultures from the initial presentation remained negative.

With the negative WBC scan, it was likely the flail anterior leaflet was due to premature valve failure, and the patient was offered the option of either a trans-catheter or open surgical mitral valve repair approach. The patient opted for a surgical mitral valve replacement. A left heart catheterization was done prior to surgery, which demonstrated normal coronary arteries. The patient underwent a redo mitral valve replacement with a redo sternotomy with a 29-mm Edward Mitris Resilia mitral valve. One of the mitral valve leaflets was found to be completely torn and did not demonstrate any evidence of infection. An intraoperative transesophageal echocardiogram confirmed a well-seated valve with no significant paravalvular leak, stenosis, or residual regurgitation.

Unfortunately, during retrosternotomy, the patient suffered an aortic laceration from a previous sternotomy wire and suffered a 17-minute circulatory arrest requiring a cardiopulmonary bypass. During the patient’s initial postoperative period, she was encephalopathic. A subsequent MRI of the brain demonstrated multiple infarcts within the central pons, thalami bilaterally, left splenium, and frontal lobes. Her post-operative hospital course was complicated by a prolonged ICU stay with mechanical ventilation and metabolic encephalopathy. After 49 days, the patient was discharged to inpatient rehab services, where she is continuing to recover and follow up with cardiology. The complication suffered during open surgical valve replacement was significant and further supports the trend towards minimally invasive valve replacement.

## Discussion

Structural valve degeneration can occur due to a number of mechanisms. These include dystrophic calcification that is prosthesis-related due to calcium phosphate deposition or recipient-related by absorbing calcium-binding proteins from the serum. Leaflet calcification can lead to increased valve stress during the cardiac cycle and lead to valve failure. Host immune activation and foreign body reactions encompass another etiology of valve failure, as activated platelets and leukocytes can adhere to the valve, leading to thrombosis and valve failure [4]. Patients with prosthetic valves are at higher risk of endocarditis, and this can lead to complications such as valve dehiscence, abscess, fistula, or myocardial infarction [5]. 

This case is unique due to the absence of masses or calcifications on the prosthetic valve on gross examination as well as the negative tagged WBC scan. Tagged white blood cell scans, also known as Indian 111-labeled WBC scans, are a type of nuclear imaging used to locate infections when other imaging modalities have not yielded any diagnostic information [6]. These tests have up to 94% sensitivity and 100% specificity in detecting infective endocarditis [7]. This case highlights the utility of this scan in ruling out infective endocarditis and identifying idiopathic prosthetic valve failure when the initial echocardiogram is negative.

This case is also uncommon due to the premature nature of valve failure four years after the operation in 2019. The St. Jude Medical Epic Heart Valve was studied in a United States Food and Drug Administration regulatory study in 22 investigational centers from 2003 to 2006. During this time, there were no structural deterioration events in patients with a bioprosthetic mitral valve (n = 204) [8]. This information makes this patient’s idiopathic structural valve failure within four years highly unusual. For many patients, eventual valve degeneration is one of the main complications, with 10-year freedoms of 78%, 89%, and 100% in the <60, 60-70, and >70 age groups at the time of valve replacement. Thus, echocardiography is recommended after five years to detect signs of valve degeneration and calcification, but this is not an effective method for preventing an idiopathic flail leaflet and acute valve failure [2]. Further studies are needed to determine if there are any worthwhile methods for identifying tissue valves at risk for idiopathic failure.

## Conclusions

Spontaneous bioprosthetic mitral valve failure is a rare complication less than five years after placement. This case demonstrates idiopathic bioprosthetic mitral valve failure four years after insertion, resulting in a flail mitral valve leaflet. As idiopathic structural failure is rare, this case also exhibits the utility of a tagged WBC scan as a tool in the diagnostic arsenal to rule out endocarditis as a cause of valve failure when transthoracic and transesophageal echocardiograms are negative.

## References

[1] Turi ZG (2004). Cardiology patient page. Mitral valve disease. Circulation.

[2] van der Merwe J, Casselman F (2017). Mitral valve replacement-current and future perspectives. Open J Cardiovasc Surg.

[3] Enta Y, Nakamura M (2021). Transcatheter mitral valve replacement. J Cardiol.

[4] Kostyunin AE, Yuzhalin AE, Rezvova MA, Ovcharenko EA, Glushkova TV, Kutikhin AG (2020). Degeneration of bioprosthetic heart valves: update 2020. J Am Heart Assoc.

[5] Khalil H, Soufi S (2022). Prosthetic Valve Endocarditis. https://www.ncbi.nlm.nih.gov/books/NBK567731/.

[6] Lewis SS, Cox GM, Stout JE (2014). Clinical utility of indium 111-labeled white blood cell scintigraphy for evaluation of suspected infection. Open Forum Infect Dis.

[7] Pucar D, William Strauss H (2022). Infective endocarditis white blood cell imaging: is there an added value to the first-line imaging?. J Nucl Cardiol.

[8] Jamieson WR, Lewis CT, Sakwa MP (2011). St Jude Medical Epic porcine bioprosthesis: results of the regulatory evaluation. J Thorac Cardiovasc Surg.

